# Subcutaneous Fat Necrosis of the Newborn: A Case Report of a Term Infant Presenting with Malaise and Fever at Age of 9 Weeks

**DOI:** 10.1155/2015/638962

**Published:** 2015-10-08

**Authors:** Ayuk Adaeze Chikaodinaka, Anikene Chukwuemeka Jude

**Affiliations:** ^1^Department of Pediatrics, University of Nigeria Teaching Hospital, PMB 01129, Enugu, Nigeria; ^2^University of Nigeria Teaching Hospital, PMB 01129, Enugu, Nigeria

## Abstract

*Background*. Subcutaneous fat necrosis (SFN) is a rare, temporary, self-limited pathology affecting adipose tissue of full-term or postmature neonates. It is a rare entity especially in Nigeria and usually occurs in the first weeks following a complicated delivery. Because it is not very common, diagnosis is easily missed. It may resolve spontaneously without sequelae but patients need to be followed up because of development of late complications especially hypercalcemia. We report a case of SFN of the newborn noted within one week of birth and highlight the need for proper prompt diagnosis and the need for follow-up to assess possible complications.

## 1. Background

Subcutaneous fat necrosis (SFN) is a rare, temporary, self-limited pathology affecting adipose tissue of full-term or postmature neonates [1, 2]. It is a rare entity and usually occurs in the first weeks following a complicated delivery [3]. Where the diagnosis is not considered and patient not followed up, complications may arise and patient may be mismanaged. Even though spontaneous resolution without sequelae is the norm, patients should be followed up for development of late complications of SFN, especially hypercalcemia. Symptoms of complications can also be missed because the initial correct diagnosis was not made. We report a case of SFN of the newborn noted within one week of birth and highlight the need for proper prompt diagnosis and the need for follow-up to assess possible complications.

## 2. Case Presentation

**Figure 1:**
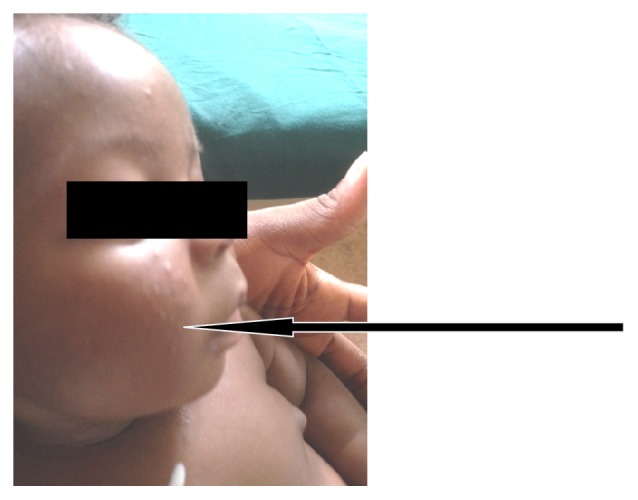
Firm nodular erythematous subcutaneous mass on the face noted on 4th day of life at the newborn nursery.

**Figure 2:**
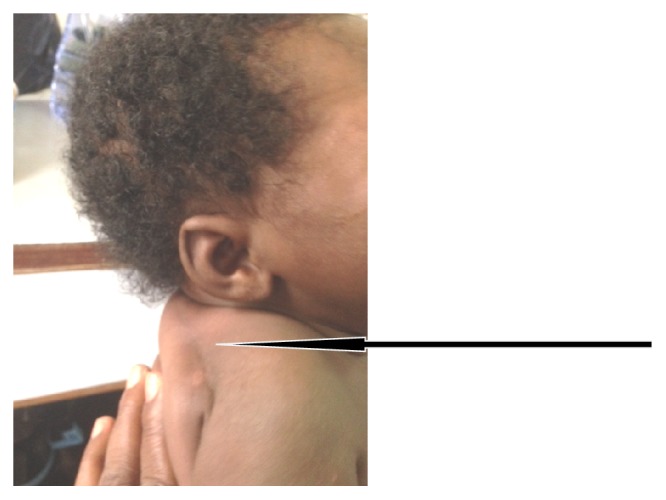
Subcutaneous erythematous nodules at nape of the neck noted in first week of life.

**Figure 3:**
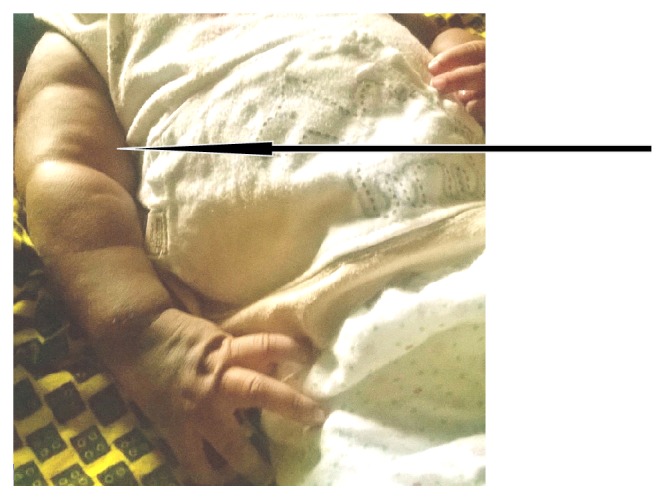
Slightly erythematous nodules noted subcutaneously within first week of life involving different parts of the body including the arms and forearms. Thus arms appear larger than they should be due to edema.

**Figure 4:**
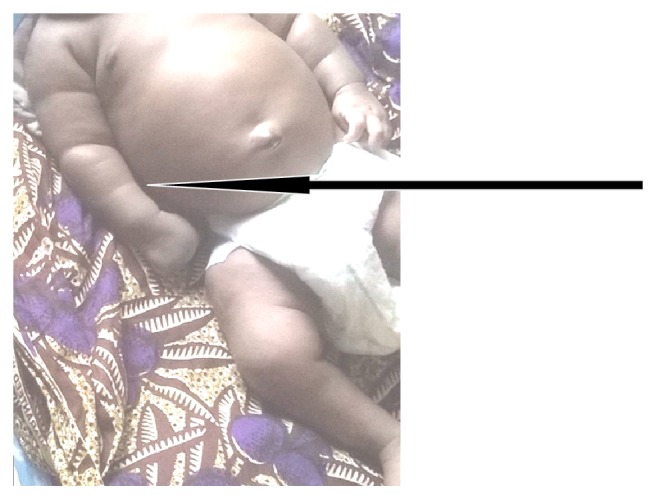
General regression in nodules and limb sizes noted at 9th week of life during emergency admission.

**Figure 5:**
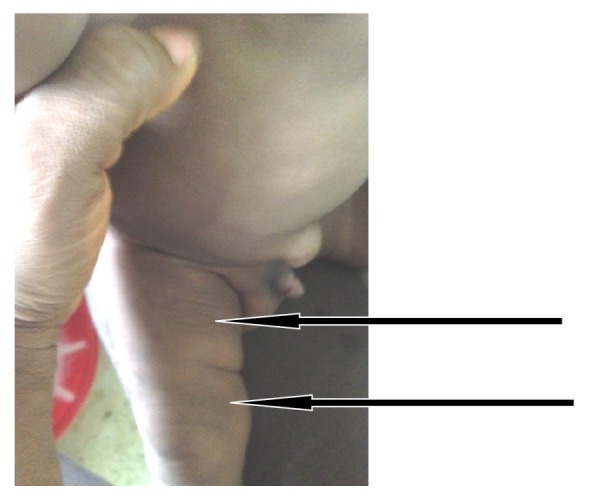
Residual hyperpigmented patch on the right thigh with small discrete swellings on the skin over anterior surface of the thighs noted at presentation to the emergency department.

Our patient is a male infant who was born term and presented to the emergency room of our hospital when he was 9 weeks old. His complaints were recurrent fever since discharge from our newborn special care unit at 1 week of birth. He also had developed recurrent vomiting that was usually postprandial with associated significant weight loss. Birth history revealed that he was macrosomic with a birthweight of 4.5 kg. He was born via caesarian section due to cephalopelvic disproportion. Other important perinatal histories were that of meconium-stained liquor, perinatal asphyxia (APGAR 4, 5, 7), and neonatal seizures on 1st day of life. During the initial newborn admission at about 4th day of life, mother noted that the patient had extensive purplish, nontender, firm subcutaneous nodules of uncertain diagnosis. The nodules were localized to the buccal fat region where they were first noted and then later spread to involve the neck, arms, forearms, thighs, and calves (Figures 1, 2, and 3). After discharge he visited several health facilities seeking help for this skin pathology including dermatology consult. During this period he was noted to be having recurrent fever and vomiting. He received several antibiotics before presenting to the emergency ward with the same complaints. At the time of presentation, most of the body swellings had regressed in size. The general appearance of the limbs with subcutaneous nodules was now at this admission less massive compared to the first week of life (Figure 4). Further examination revealed an axillary temperature of 38.1°C, slightly depressed anterior fontanelle but normal skin turgor. He still had residual facial nodular swelling that was flat, firm, attached to overlying skin, reddish, and measured about 4 cm in its widest diameter. Some of the child's initial nodules that were noted in the first week of life had substantially regressed and were now only noted as areas of hyperpigmentation as was seen on the skin of the right thigh measuring about 1 cm by 2 cm (Figure 5). His weight loss was evidenced by loose skin folds (arm and thigh) and a weight drop from previously documented 6.1 kg to current weight of 5.3 kg. A complete blood count showed a haemoglobin level of 10.2 g/dL, total WBC 18,300 (neutrophil 48%, lymphocytes 47%, monocytes 2%, and eosinophils 3%). The total platelet count was 534,000 and he had an elevated total ionized calcium level (iCa) of 1.72 mmol (1.12–1.32). A dipstick urinalysis showed nitrite +, leucocyte ++ which suggested a possible urinary tract infection. A follow-up urine culture was not done as child had been on several antibiotics up to the time of this admission. The routine kidney function results were within normal limits. Immediately following admission into emergency room, dehydration was corrected and parenteral broad spectrum antibiotics commenced. On the 4th day of admission on obtaining the initial calcium result, steps to manage the hypercalcemia were taken. Formula feeding was initially stopped while continuing breast feeding; he was commenced on intravenous fluid hydration therapy of 10% dextrose in 0.45% saline combined with diuretic therapy for 48 hours. The iCa level repeated thereafter was 1.65 mmol and after 2 weeks had dropped further to 1.25 mmol. Skin lesions continued to regress and he continued to do well clinically. With the normalization of calcium levels and the good clinical progress further calcium assays were not continued. He was being followed up in our outpatient clinic at the time of this report.

## 3. Discussion 

Subcutaneous fat necrosis of the newborn (SFN) is a rare form of panniculitis, an inflammation of the subcutaneous and adipose tissue [1]. It typically affects newborns. It is also known as adiponecrosis subcutanea [2]. It is a very rare disorder with no gender predilection [3]. It usually occurs in the first several weeks of life as was seen in our index patient. The exact aetiopathogenesis is unknown but postulations have been made as to the possible causes. A common theory is that stress such as that occuring from birth asphyxia in the newborn with immature fat cells induces inflammation, solidification, and necrosis. This leads to the formation of granulomatous infiltrates [3]. Histology of these granuloma have shown increased expression of 1-alpha hydroxylase known to activate vitamin D3 [4, 5]. The increased activity of vitamin D3 causes increased release of calcium. This could account for the hypercalcemia usually seen in SFN [3].

Another speculation for hypercalcemia is as observed in other conditions that have granulomatous lesions such as sarcoidosis [6]; the presence of granulomatous skin lesions is thought to be a source of extrarenal production of 1,25-OH vitamin D (calcitriol). The calcitriol causes increased intestinal calcium absorption and hence the resultant hypercalcemia [7]. In SFN unregulated production of 1,25-dihydroxyvitamin D by the granulomatous cells of fat necrosis could also result in hypercalcemia as reported by some authors [5, 8]. Furthermore, the combination of hypercalcemia, normal serum concentration of 25-hydroxyvitamin D, elevated 1,25-dihydroxyvitamin D, and a suppressed parathyroid hormone would indicate an abnormal 1,25-dihydroxyvitamin D production with possible increased intestinal absorption of calcium [8]. Unfortunately due to limited finances the 1,25-dihydroxyvitamin D and parathyroid hormone were not assayed and these would have shed further light on the possible sources of hypercalcemia in our index patient.

Susceptible children who may have to undergo body cooling for the management of perinatal asphyxia could also develop subcutaneous necrosis of fat and adipose tissue [4]. Our patient was exposed to stress from birth asphyxia even though he did not receive body cooling therapy. SFN typically occurs in a full-term newborn as was our index patient. The skin lesions usually appear from about day four after delivery [9] and have been associated with certain predisposing factors such as obstetric trauma, meconium aspiration, asphyxia, hypothermia, or peripheral hypoxemia [3]. Many of these factors were present as antecedent clinical history in our patient and may have predisposed our patient to SFN. There has also been a putative report of macrosomia. Mahé et al. [9], in a systematic review of risk factors, clinical manifestations, complications, and outcome in 16 affected children over a 6-year period, reported macrosomia in about half of the children studied. Our patient was also macrosomic with a birthweight of 4.5 kg.

The typical skin lesions appear as erythematous to purplish, firm subcutaneous nodules [10] and are usually asymptomatic. They may appear on the cheeks, buttocks, back, thighs, or upper arms and may be focal or extensive. Sometimes they may be tender during the acute phase [11], as was seen in 4 of the children reviewed by Mahé et al. [9]. Lesions were first noted in the buccal fat region in our patient before spreading to involve other parts of the body.

Among symptoms our patient presented with was persistent fever. Shumer et al. [12] noted fever in 57% of the cases they studied. They postulated this to be due to elevated levels of prostaglandin E2 found in some of the SFN patients with hypercalcemia as well as elaborations of interleukin-1 from the granulomas of SFN [12]. Our patient had elevated calcium levels and this may be the plausible cause of his recurrent fever.

We could also attribute the fever to the possible urinary tract infection as comorbidity which patient had. It may also be part of the symptom complex of SFN. Occurrence of hypercalciuria secondary to hypercalcemia especially in patients diagnosed with rheumatic disease and having a renal comorbidity may lead to sterile leukocyturia [13]. This could mimic a urinary tract infection [13]. In established leukocyturia, however, WBC count is usually more than 15 WBC per hpf [14]. The possibility of UTI as a comorbidity was considered in this patient who had lower WBC of between 5 and 10 WBC per hpf and nitrites in urine. The urinary calcium excretion rate was however not done to further confirm the presence of calcium in urine but the patient had normal kidney function. Elevated levels of serum calcium have been noted as a rare complication of SFN and usually occur with disease regression [15, 16]. Symptoms of hypercalcemia include lethargy, vomiting, poor feeding with attendant weight loss, polyuria, and fever [17]. Severe elevations will lead to nephrocalcinosis with progressive reduction in renal function [17]. Screening for hypercalcemia in children with a possible diagnosis of SFN is therefore important to reduce likelihood of morbidity from hypercalcemia [3]. This requires a high index of suspicion on the part of the attending physician to think early of the diagnosis and know when best to assay for calcium levels. Hypercalcemia occurs as the skin lesions begin to regress and this may coincide with the time when our patient developed fever. The symptom of recurrent vomiting in our patient may also have been part of the symptom complex of hypercalcemia. He also had seizures on day one of life. This may be more attributable as a sequel of birth asphyxia rather than high calcium levels as hypercalcemia is uncommon at that stage of SFN.

The presence of hypercalcemia may also be asymptomatic. Shumer et al. [12] also noted in their study population that 43% of patients with severe hypercalcemia (≥3 mmol/L) were asymptomatic [12].

Apart from clinical diagnosis, SFN can be further confirmed by histopathology following a tissue biopsy, which is an invasive procedure. When the carefully collected biopsy is subjected to histopathology, the following are diagnostic: radially arranged clefts of crystalline triglyceride within fat cells, granulomatous cellular infiltrate composed of lymphocytes which confirms fat necrosis, and presence of histiocytes, multinucleated giant cells, and fibroblasts [11]. Our patient did not get the benefit of histopathology due to the invasive nature of tissue biopsy.

SFN usually runs a self-limiting course [3]. When our patient presented to the emergency room, most of the skin lesion had already regressed. However this also coincides with the period of possible complications of hypercalcemia as was seen in our patient. This could be easily missed if the diagnosis of SFN is not considered. Treatment should aim at preventing and managing the complications of hypercalcemia when present. Hypercalcemia has been successfully managed by a combination of diet modification, fluids, and drugs. Diet modification will include low calcium formula [12]. A combination of saline fluid hydration with calcium wasting diuretics is a recognized standard first-line intervention [18]. Corticosteroids and bisphosphonates may be used for further management when adequate reduction in calcium levels is not obtained with the first-line intervention [3]. Samedi et al. [19], in a case report, noted that the use of pamidronates (a bisphosphonate) had a faster onset of action than corticosteroids and thus advocated it as a possible first-line management of SFN with very severe hypercalcemia. Similarly Alos et al. [20] in a case series involving four infants found that after initial trial of hydration and diuretics and there was not much reduction in iCa levels there was documented good outcome with the use of pamidronates. Our patient however did well with diet modification, optimal hydration, and the use of diuretics.

The possible differential diagnoses to be considered alongside SFN include skin infections in the new born such as bacterial cellulitis, erysipelas, CMV infection, sclerema neonatorum, steroid-induced fat necrosis, deep infantile hemangioma, dermohypodermitis, neurofibromatosis, lipogranulomatosis (Farber disease), sarcomas including pediatric rhabdomyosarcoma, and other panniculitides [3, 21]. The patient however had distinctive features that pointed most to the diagnosis of subcutaneous fat necrosis of the newborn.

As part of management of these babies who have SFN with raised calcium levels, it is recommended that at least biweekly follow-up visits are done for up to a period of 6 months [3, 22]. This is to monitor for manifestations of any complications of hypercalcemia and be able to intervene appropriately. Calcium assay was repeated at 2 weekly intervals in our index patient until normal values were obtained and follow-up medical check-ups were continued at the outpatient clinic.

## 4. Conclusion

Subcutaneous fat necrosis is a rare finding in our environment and can present with complications such as hypercalcemia. Presenting this case highlights the need for a high index of suspicion for the medical personnel, to aid early diagnosis and appropriate intervention. If correct diagnosis is made and child is properly followed up, the possible complications arising from hypercalcemia can be prevented or properly managed. Follow-up following resolution of skin lesions is also emphasized. This will help in reducing morbidity or mortality from SFN in the newborn.

## Abbreviations

SFN: Subcutaneous fat necrosis.
